# Transmission of biology and culture among post-contact Native Americans on the western Great Plains

**DOI:** 10.1038/srep25695

**Published:** 2016-08-12

**Authors:** Stephen J. Lycett, Noreen von Cramon-Taubadel

**Affiliations:** 1 Department of Anthropology, University at Buffalo, SUNY, 380 MFAC-Ellicott Complex, Amherst, NY 14261, USA

## Abstract

The transmission of genes and culture between human populations has major implications for understanding potential correlations between history, biological, and cultural variation. Understanding such dynamics in 19th century, post-contact Native Americans on the western Great Plains is especially challenging given passage of time, complexity of known dynamics, and difficulties of determining genetic patterns in historical populations for whom, even today, genetic data for their descendants are rare. Here, biometric data collected under the direction of Franz Boas from communities penecontemporaneous with the classic bison-hunting societies, were used as a proxy for genetic variation and analyzed together with cultural data. We show that both gene flow and “culture flow” among populations on the High Plains were mediated by geography, fitting a model of isolation-by-distance. Moreover, demographic and cultural exchange among these communities largely overrode the visible signal of the prior millennia of cultural and genetic histories of these populations.

It is evident that historical (long term) patterns of divergence and descent can be created in both the genetic and cultural systems of human communities given that both are based on systems of inheritance, which facilitates some degree of continuity^1^,^2^. Equally, however, it is apparent that when people from diverse historical lineages come into contact, so their genes and cultures collide, potentially leading to genetic and/or cultural admixture^3^,^4^. These various sets of processes make understanding links between historical factors and correlations between genetic patterns and cultural patterns in humans a challenge. And yet, studies that help better understand these dynamics and their effects in empirical contexts are essential if we are to approach this challenge. So far, however, surprisingly few studies of human biological and behavioral variation have directly examined the congruence of among-group patterns of variation in biological and cultural data sets. Most studies to date focus on correlations between genetic and linguistic data (e.g.,^5^,^6^,^7^). These studies have shown that at a broad geographic level, there is often co-correlation of genetic and linguistic lines of divergence and descent. One rare example of a study examining non-linguistic examples of culture alongside genetic data for the same communities in Africa^8^ identified that cultural traits and genetic patterns coincide because of transmission down vertical (familial) lines for both sets of traits at a continental level. On a more localized level, it has been shown that traditional song characteristics among indigenous populations of Taiwan correlate with mitochondrial DNA patterns for the same populations, while linguistic data were also shown to correlate with the genetic patterns^9^. The authors accordingly suggest their results indicate an historical signal of diverging coevolution between song characteristics and genetics. However, given these examples it would also seem important to examine the extent of co-correlation between biological and cultural pathways of transmission in a situation where the known historical dynamics are likely to involve higher degrees of genetic and cultural admixture, to determine what might be inferred under these contrasting conditions.

Native American communities that occupied the western portion of the Great Plains during the post-contact (historical) period have come to be globally famous^10^. These communities of horse riding, buffalo hunting, tipi-dwelling people have often in fact been held up as the archetypal image of what it is to be Native American^11^, and yet ironically, such a lifestyle was in many respects atypical and temporally restricted, owing its existence to a unique set of historical circumstances^12^,^13^. The western Great Plains (often referred to as the “High Plains” or “mixed/shortgrass Plains”) is a semi-arid, grassland region, which until reduction of their numbers to the point of near extinction in the late 1800s, supported large herds of American bison (*Bison bison*)^14^. This western portion of the Great Plains runs north to south from southern Canada (Alberta/Saskatchewan) to western Texas, and east to west from approximately the 100th meridian to the Rocky Mountains. The introduction of the horse to the region in the 1700s, as well as an increasing number of firearms, created new opportunities for a lifestyle based around the hunting of buffalo, and new, linguistically diverse groups of people were subsequently being drawn onto the plains at this time^15^. Migrations of (largely) new, linguistically and biologically diverse groups of people to the region were, however, also precipitated by other events. In particular, the inflow of Europeans in the east of the continent led to a displacement of many tribes, increasing agitations between indigenous peoples and leading to a chain reaction of movement across the continent, ultimately adding to the influx of new peoples^13^,^15^.

These combined push and pull effects ensured that by the 1800s, communities from at least five different language families were occupying the western Great Plains (Fig. 1), pursuing a nomadic, equestrian lifestyle formulated around an economy based on buffalo hunting. Perhaps more remarkably, these historically diverse groups of people came to share many visible aspects of their cultural practices, thus leading to the formation of what has often been referred to as the “Great Plains culture”^10^,^16^,^17^. Hence, cultural features shared in common at this time included not only an equestrian lifestyle based around buffalo-hunting, but also practices such use of tipis, lack of pottery production, adoption of new ceremonial practices such as the “Sun Dance,” ritualized combat, and elaborated decoration of rawhide and skins, especially via painting, beadwork, and quillwork^18^. While these elements are by no means unique to the High Plains, in combination they mark a distinctive historico-geographic phenomenon, all the more remarkable given the linguistically and historically diverse backgrounds of the communities concerned. Indeed, Goddard^19^, p. 61 aptly summarized these dynamics when he noted:

“linguistic diversity was much greater than the diversity of other aspects of their cultures and must be a retention from a time when they had less contact with each other and were culturally more distinct … The general picture that is suggested is of diverse peoples retaining their distinct ancestral languages while adopting new and to a large extent shared lifeways after coming into contact with each other.”

Examining potential congruence between cultural diversity and genetic patterns and their pathways of transmission under such dynamic and historically contingent conditions obviously presents a major challenge. Moreover, in this specific case, additional analytical problems are present. Most obviously, there is the passage of time between the late 19th century and the present day, meaning we are dealing with historical rather than directly contemporary populations. Furthermore, there is currently an absence of contemporary genetic data for the living descendants of the equestrian bison-hunting populations of the past^20^, which might assist in this endeavor. The passage of time, however, means that even if such data were available, it would be for individuals born a century or more after those who lived during the historical events of interest here. Indeed, given recent repatriation, many historical biological data are no longer available for study.

Here, we sought to overcome these challenges and simultaneously examine both biological and cultural pathways of transmission in nine ethno-linguistic tribes who occupied the western Great Plains during the 19th century (Fig. 1). More specifically, we also aimed to determine if these two inheritance systems shared similar pathways of transmission. To do so, we collated cultural and biometric data common to all nine tribes. The cultural (ethnohistorical and artifactual) dataset comprised differences in behaviors across tribes, relating to features associated with their performance of the religious “Sun Dance” ceremony, moccasin decorative variants, parfleche (rawhide bag) design features, and tipi design (Fig. 1). The biometric data comprised cephalometric variables taken from living individuals, collected *circa* 1888–1903 under the direction of Franz Boas^21^, and represent craniofacial shape variables (see Methods). Such data were used because craniofacial variables have been shown to vary according to a neutral evolutionary model in human populations^22^ facilitating their use as a proxy for among-group neutral genetic variation in cases where genetic data are unavailable^23^,^24^,^25^,^26^.

In a situation such as the historical Great Plains, geographic distance between communities provides a primary hypothesis for predicting both cultural and biological similarities and differences between those groups. This is because under such circumstances, geographic distance is likely to mediate likelihood and intensity of between-group interactions, whereupon similarities and differences in cultural and/or genetic patterns are, most parsimoniously, simply a function of mutation (copying and transmission errors), drift (stochastic trait loss), migration (between group movements of people), and/or diffusion (exchange of cultural ideas without demographic exchange). In other words, under these conditions, what has long been referred to as “isolation by distance” in population genetics parlance^27^, provides a null model that predicts a relationship between geographic distances and biological distances among groups, as well as between geographic distances and cultural distances among groups^28^,^29^. Hence, we first independently assessed goodness-of-fit for among-tribe biological and cultural distances to the isolation-by-distance model using matrix correlation statistics. Biometric and cultural data were also examined for correlation against a matrix describing the linguistic affinities of the tribes, which in the case of the Plains tribes can be seen as a measure of deeper historical relationships among tribes, independent of the recent arrival of most of these groups to the region^19^. Moreover, biometric and cultural data were also compared with a matrix representing documented patterns of alliance and hostility among tribes during the late 19th century. We also tested whether among-tribe cultural and biological patterns of variation correlate with each other.

## Results

No statistical evidence was found for a correlation between the geographic distances among tribes and linguistic distances among them (r = −0.230, p = 0.102). This result thus accords with the known historical situation, in that many groups were recent migrants to the region and hence their geographic position, and the geographic relationships between tribes, had nothing to do with deeper historical factors as measured by linguistic affinity.

With respect to tests of our central hypotheses, both the biological and cultural data sets independently showed a statistical fit to the isolation-by-distance model (Table 1). That is, both the cultural datasets and the biometric data for tribes demonstrated a significant correlation with the matrix representing geographic distances between them (Table 1). The biometric data displayed no evidence of a statistically significant correlation with either language distances or the model matrix of intertribal political relations (Table 1). Likewise, cultural patterns of variation were not significantly correlated with the matrix of language distances. Hence, the analyses indicate that both cultural and biometric patterns of among-group variation were mediated by geographic distances among tribes in accordance with the isolation-by-distance null model. However, a significant relationship between inter-tribal political dynamics and cultural variation (Table 1) suggest that such factors had an influence on the eventual patterns of differentiation among groups, once cultural variants had been transmitted.

We also tested whether among-tribe cultural patterns of variation correlated with among-tribe biometric variation directly. We found that among-tribe cultural and biometric patterns of variation were not significantly correlated with each other (r = −0.138; p = 0.267).

## Discussion

Here, we have examined the extent to which geographic distances among Native American communities occupying the western Great Plains during the 19th century mediated patterns of transmission in both their genetic and cultural inheritance systems. Our central results identify that among-tribe patterns of cultural and biometric variation correlate with the geographic distances among these tribes. In other words, our results identify that both gene flow and “culture flow” among these communities was mediated partly by the geographic distances between pairs of tribes in any given instance.

Our results also show that deeper, historical patterns of cultural and biometric differentiation among these tribes, as measured by correspondence with their ancestral languages, were largely overridden by their more recent geographically mediated interactions, which led to genetic admixture and exchange of cultural ideas. Admittedly, use of direct genetic data for these historical populations rather than necessary use of proxy biometric data, may have revealed stronger evidence of deeper historical signal in terms of biological patterns of differentiation. Nevertheless, our results indicate that recent historical events of population exchange among these groups led to empirically recognizable effects in the available data in a relatively short time span. Indeed, there are two ways in which “culture flow” may occur: either via demographic exchange between populations or by transferal of ideas in the absence of demographic exchange^30^. Our result demonstrating that biometric variation fits a model of isolation by distance suggests that demographic exchange—not simply exchange of ideas—was an important vector of cultural transmission among tribes. However, inter-community conflict and political alliances among tribes occupying the western Great Plains is well noted^31^,^32^. The significant relationship between inter-tribal political relationships and cultural variation also suggest that these dynamics had an influence on the eventual patterns of differentiation among groups once cultural variants had been transmitted. Indeed, subsets of the cultural data used here (parfleche features) have previously been shown to fit a pattern suggestive of influences of this type^33^.

Linguistic differences among the various groups appear to have provided little barrier to either gene flow or culture flow among groups, at least such that these led to a deviation from patterns that can be predicted on the basis of geographic distances among tribes alone. Such a pattern is explicable, however, when it is considered the many of the cultural variants we examined here (moccasin decorative features and parfleche characteristics in particular) were the product of female craftswomen. Given that girls and women were taken as captives by tribes hostile toward one another, as well as married into allied tribes^31^,^34^, the role that females played in producing both the biometric and cultural patterns displayed in our results is thrown into sharp focus. Equally, however, our results highlight the rapid effect that genetic and cultural interchange can potentially have on the biometric patterns and measurable cultural attributes of groups, which on the basis of linguistic distinctions, are known to have had distinct historical divergences over a longer time frame.

It should be noted that these findings have been generated within a framework of analysis that could be considered broadly “evolutionary” in scope, in respect to the analysis of both biometric patterns *and* cultural patterns (i.e., within a framework focusing on understanding historical patterns via a process of descent with modification). In this sense, our analytical framework shares much in common with those who have previously advocated such an approach on the basis of identifying broad and deep historical correlations between genetic, linguistic, and/or other cultural dimensions of human variability (e.g.,^5^,^6^,^7^,^8^). Although the situation we have examined here is one in which both gene flow and culture flow between groups has been prevalent, even to the extent of obscuring deeper historical patterns that might otherwise have been visible, our analyses also highlight the value of an evolutionary framework rather than being cause to incite its abandonment. Rather, what these results reiterate is the need for an evolutionary framework for understanding both links and divergences between human biological and cultural patterns, which can operate at varying scales of analysis; from the broad-scale, where deep historical divergence may be in evidence, to the local, more micro-scale level, where cultural and genetic exchange may be causing the evolution of alternative patterns in both types of data.

## Materials and Methods

### Tribes examined

**Figure 1:**
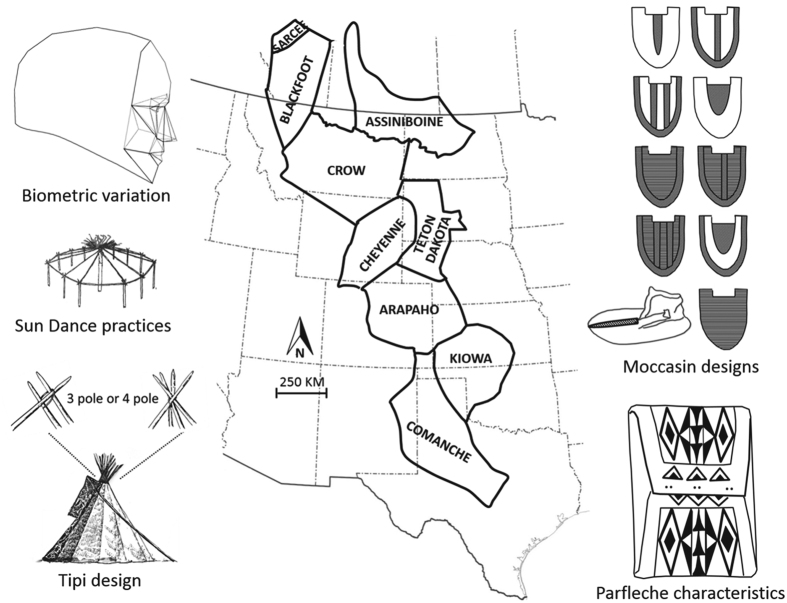
Schematic depiction of the late nineteenth century geographic distributions of the nine ethno-linguistic tribes considered in our analyses, alongside illustrations representing the combination of cultural and biometric data we use in our study. The nine tribes are distributed from present-day Texas in the south (Comanche) to Canada in the north (Sarcee, Blackfoot, Assiniboine). (Figure drawn by the authors, modified and redrawn after the originals contained in refs 10,29,47).

A total of nine tribal groups were examined here for which common cultural and biometric data were available (Fig. 1). These tribes are among those that most extensively adopted equestrian nomadism centered on a buffalo hunting economy, with accompanying cultural features that came to exemplify tribes of the western Great Plains during the 18th and 19th centuries^10^,^17^.

### Biometric data

The Franz Boas dataset comprises anthropometric and cephalometric (biometric) measurements for several thousand individuals from North American tribes, which were recorded on living individuals between 1888 and 1903^21^. The dataset used here comprised biometric measurements of the head and face: head length, head breadth, facial height, facial breath, nasal height, and nasal breadth. Cephalometric data are directly comparable to underlying homologous craniometric dimensions, which multiple previous studies have shown provide a reliable proxy for underlying patterns of neutral genetic covariation in human populations (e.g.,^35^,^36^,^37^,^38^,^39^,^40^). Global patterns of shape variation in the human skull have been shown to fit a model of mostly neutral diversification, indicating that non-neutral forces such as directional selection have played a relatively small role in generating among-population diversity patterns^22^. Hence, in cases where direct genetic evidence is unavailable, craniometric data are often employed as a useful proxy for the underlying population history of population divergence, migration, and gene flow (e.g.,^23^,^24^,^25^,^26^). Moreover, Konigsberg and Ousley^41^ utilized a subset of the Boas database (including the variables used here) for which pedigree information (i.e., knowledge of close familial relationships among individuals) was available to assess the congruence of the underlying additive genetic (G) matrix and the observed phenotypic covariance (P) matrix. They found that G and P were proportional and that among-tribe additive genetic and phenotypic distances were strongly congruent. On the basis of these results, they argued that the Boas dataset should be used in further analyses of population history, particularly where genetic data are unavailable. To eliminate potentially confounding effects of variation due to ontogeny and sex, only data from adult males were utilized (Table 2). To correct for potential systematic differences in the isometric scaling of individual groups, which could mask patterns of among-group biometric differentiation, each biometric variable was divided by the geometric mean of all variables for that individual^42^,^43^. A pairwise among-group biometric distance matrix was generated using the quantitative genetic model of Relethford and Blangero^44^, under the conservative assumption of complete heritability and correcting for sampling bias following ref. 45 (Supplementary Table 1). On average, individuals represented in each population were born between 1850 and 1875 (Table 2, where average year of birth = average age of population sample, subtracted from years of data collection, i.e., 1888–1903). Although there are discrepancies in sample size in the raw data (Table 2), a Euclidean distance matrix based on sample size differences among tribes showed no significant relationship with biometric patterns among tribes (r = −0.197, p = 0.33).

### Cultural data

The ethnographic and artifactual cultural dataset was collated from several published sources^46^,^47^,^48^,^49^ and processed according to procedures previously utilized elsewhere^29^,^33^. The cultural features were comprised of behavioral variations among tribes in performance of the religious Sun Dance ceremony (*n* = 82 traits), moccasin decorative features (*n = *10 traits), parfleche design features (*n* = 23 traits), and a single trait associated with tipi design (3 pole or 4 pole base). Traits were typically recorded on a presence/absence basis, although three trait variants relating to parfleche design were treated as categorical variables. An among-tribe cultural distance matrix (Supplementary Table 2) was computed using the open source software PAST^50^. PAST facilitates the creation of a distance matrix using mixed data types, which are combined using an average that is weighted by the number of variates of each type. Presence/absence data were computed using Jaccard distances. Jaccard measures are particularly suitable for binary data, because focusing on differences in shared presences rather than absences, controls for absences that may simply be the result of observational bias^51^,^52^. Distances for the categorically coded variables were computed using the Hamming distance. In the case of the Sun Dance data, some traits were originally coded as missing (?) in the published source^48^, and data for the Comanche were also absent for the Sun Dance ceremony. Conservatively, missing data were treated by pairwise deletion in computing the among-tribe distances; that is, if a data point was coded as missing for one of the variables in a pair, that variable was omitted when describing total distance between those two groups.

### Computing geographic distances

The geographic positions and extent of tribal ranges *circa* the mid-late 19th century were taken from ref. 10, who notes that the map is, inevitably, somewhat a schematic approximation of relative tribal territories. However, when central points within these approximations are treated as point coordinates (as here) about which groups moved and redistributed themselves, they facilitate spatial analysis. That is, center points of these approximations, at a scale of the entire region, reflect the major aspects of spatial distances among groups during the latter part of the 19th century. Geographic coordinates at the center of each territory (Supplementary Table 3) were used to compute a geographic distance matrix using open source software published by the American Museum of Natural History^53^.

### Linguistic affinities

The linguistic affinities of the nine tribes considered here are well known and consistently documented^19^,^54^,^55^,^56^. Linguistic affinities of the nine tribes were converted into a distance matrix (Supplementary Table 4), quantitatively representing the relative linguistic similarity/dissimilarity among all groups. The method utilized broadly follows that of ref. 57 (for similar application see ref. 51), whereupon documented linguistic affinities were assigned the following scores: 5% for groups that spoke languages from entirely different families; 50% for groups that spoke a language from the same family but belonging to different subgroups; and, 80% for groups speaking languages of the same subgroup within a family.

### Intertribal political relationships

Major patterns of known hostility (i.e., intertribal conflict) and alliance as they stood during the latter decades of the 19th century are reasonably well documented^31^,^33^. A model matrix was thus created which represented whether tribes were either politically allied or openly hostile to each during the latter part of 19th century (Supplementary Table 5).

### Matrix correlation statistics

Correlations between distance matrices were examined for statistical correlation. Since matrices violate the assumptions of traditional correlation statistics, matrices were analyzed using Mantel tests^58^,^59^ where upon statistical significance (α = 0.05) is determined via permutations of the original data (here, involving 10,000 randomizations). All correlation analyses were undertaken using the open source software PASSaGE 2^60^.

## Additional Information

**How to cite this article**: Lycett, S. J. and von Cramon-Taubadel, N. Transmission of biology and culture among post-contact Native Americans on the western Great Plains. *Sci. Rep.* **6**, 25695; doi: 10.1038/srep25695 (2016).

## Supplementary Material

Supplementary Information

## Figures and Tables

**Table 1:** Results of matrix correlation analyses (significant values in bold).

	Geographic distances *r*/*p*	Language *r*/*p*	Inter-tribal relations *r*/*p*
Cultural data	0.326/**0.039**	−0.227/0.102	−0.433/**0.012**
Biometric data	0.382/**0.038**	−0.225/0.083	0.030/0.481

**Table 2:** Description of biometric data used.

Tribe	Sample size (*N*)	Average year of birth
Arapaho	62	1854‒1869
Assiniboine	26	1851‒1866
Blackfoot (Blood/Piegan)	60	1856‒1871
Cheyenne	32	1858‒1873
Comanche	82	1857‒1872
Crow	299	1851‒1866
Teton Dakota	622	1850‒1865
Kiowa	101	1856‒1871
Sarcee	12	1860‒1875

Tribal names in parentheses indicate the names used in the Boas database, where different from the nomenclature used in the present study.

## References

[b1] Cavalli-Sforza L. L., Menozzi P. & Piazza A. The History and Geography of Human Genes. Princeton University Press (1994).

[b2] Durham W. H. Applications of evolutionary culture theory. Annu. Rev. Anthropol. 21, 331–355 (1992).

[b3] Rosenberg N. A. . Genetic structure of human populations. Science 298, 2381–2385 (2002). 12493913 10.1126/science.1078311

[b4] Jordan P. Technology as Human Social Tradition: Cultural Transmission among Hunter-Gatherers. University of California Press (2015).

[b5] Cavalli-Sforza L. L., Piazza A., Menozzi P. & Mountain J. Reconstruction of human evolution: bringing together genetic, archaeological, and linguistic data. Proc. Natl Acad. Sci. USA 85, 6002–6006 (1988). 3166138 10.1073/pnas.85.16.6002 PMC281893

[b6] Chen J., Sokal R. R. & Ruhlen M. Worldwide analysis of genetic and linguistic relationships of human populations. Hum. Biol. 67, 595–612 (1995). 7649533

[b7] Belle E. & Barbujani G. Worldwide analysis of multiple microsatellites: language diversity has a detectable influence on DNA diversity. Am. J. Phys. Anthropol. 133, 1137–1146 (2007). 17506490 10.1002/ajpa.20622

[b8] Hewlett B. S., de Silvestri A. & Guglielmino C. R. Semes and genes in Africa. Curr. Anthropol. 43, 313–321 (2002).

[b9] Brown S. . Correlations in the population structure of music, genes and language. Proc. R. Soc. B. 281, 20132072 (2014). 10.1098/rspb.2013.2072 PMC3843827 24225453

[b10] DeMallie R. J. Handbook of the North American Indians: Plains (Vol. 13). Smithsonian Institution (2001).

[b11] Ewers J. C. The emergence of the Plains Indian as the symbol of the North American Indian. Annual Report for the Smithsonian Institution 1964, 531–544 (1965).

[b12] Haines F. The Plains Indians. New York: Crowell (1976).

[b13] Hämäläinen P. The rise and fall of Plains Indian horse cultures. The Journal of American History 90, 833–862 (2003).

[b14] Lott D. F. American Bison: A Natural History. University of California Press (2002).

[b15] Holder P. The Hoe and the Horse on the Plains. University of Nebraska (1970).

[b16] Kroeber A. L. Cultural Areas and Natural Areas of Native North America. University of California Press (1947).

[b17] Wissler C. The American Indian. Oxford University Press (1938).

[b18] Penney D. W. (ed). Art of the American Indian Frontier. Detroit Institute of Arts (1992).

[b19] Goddard I. In Handbook of North American Indians: Plains (Vol. 13) ed DeMallie R. J. 61–70 Smithsonian Institution (2001).

[b20] Reich D. . Reconstructing Native American population history. Nature 488, 370–374 (2012). 22801491 10.1038/nature11258 PMC3615710

[b21] Jantz R. L. Franz Boas and Native American biological variability. Hum. Biol. 67, 345–353 (1995). 7607632

[b22] von Cramon-Taubadel N. Evolutionary insights into global patterns of human cranial diversity: population history, climatic and dietary effects. J. Anthropol. Sci. 92, 43–77 (2014). 24038629 10.4436/jass.91010

[b23] Pinhasi R. & von Cramon-Taubadel N. Craniometric data supports demic diffusion model for the spread of agriculture into Europe. PLoS ONE 4, e6747 (2009). 19707595 10.1371/journal.pone.0006747 PMC2727056

[b24] Hubbe M., Neves W. A. & Harvati K. Testing evolutionary and dispersion scenarios for the settlement of the New World. PLoS ONE 5, e11105 (2010). 20559441 10.1371/journal.pone.0011105 PMC2885431

[b25] von Cramon-Taubadel N. & Pinhasi R. Craniometric data support a mosaic model of demic and cultural Neolithic diffusion to outlying regions of Europe. Proc. R. Soc. B 278, 2874–2880 (2011). 10.1098/rspb.2010.2678 PMC3151708 21345869

[b26] Reyes-Centeno H., Hubbe M., Hanihara T., Stringer C. & Harvati K. Testing modern human out-of-Africa dispersal models and implications for modern human origins. J. Hum. Evol. 87, 95–106 (2015). 26164107 10.1016/j.jhevol.2015.06.008

[b27] Wright S. Isolation by distance. Genetics 28, 114–138 (1943). 17247074 10.1093/genetics/28.2.114 PMC1209196

[b28] Relethford J. H. Isolation by distance, linguistic similarity, and the genetic structure on Bougainville Island. Am. J. Phys. Anthropol. 66, 317–326 (1985). 3985140 10.1002/ajpa.1330660309

[b29] Lycett S. J. Dynamics of cultural transmission in Native Americans of the High Great Plains. PLoS ONE 9, e112244 (2014). 25372277 10.1371/journal.pone.0112244 PMC4221622

[b30] Clarke D. L. Analytical Archaeology. Methuen (1968).

[b31] Ewers J. C. Intertribal warfare as the precursor of Indian-White warfare on the Northern Great Plains. The Western Historical Quarterly 6, 397–410 (1975).

[b32] McGinnis A. R. Counting Coup and Cutting Horses: Intertribal Warfare on the Northern Plains 1738–1889. Cordillera Press (1990).

[b33] Lycett S. J. Differing patterns of material culture intergroup variation on the High Plains: a quantitative analysis of parfleche characteristics vs. moccasin decoration. Am. Antiq. 80, 714–731 (2015).

[b34] Moore J. H. In Archaeology, Language, and History: Essays on Culture and Ethnicity ed Terrell J. E. 31–56 Bergin & Garvey (2001).

[b35] 3Relethford J. H. Craniometric variation among modern human populations. Am. J. Phys. Anthropol. 95, 53–62 (1994). 7527996 10.1002/ajpa.1330950105

[b36] Relethford J. H. Global patterns of isolation by distance based on genetic and morphological data. Hum. Biol. 76, 499–513 (2004). 15754968 10.1353/hub.2004.0060

[b37] Roseman C. C. Detecting interregionally diversifying natural selection on modern human cranial form by using matched molecular and morphometric data. Proc. Natl Acad. Sci. USA 101, 12824–12829 (2004). 15326305 10.1073/pnas.0402637101 PMC516480

[b38] Harvati K. & Weaver T. D. Human cranial anatomy and the differential preservation of population history and climate signatures. Anat. Rec. 288, 1225–1233 (2006). 10.1002/ar.a.20395 17075844

[b39] Smith H. F. Which cranial regions reflect molecular distances reliably in humans? Evidence from three‐dimensional morphology. Am. J. Hum. Biol. 21, 36–47 (2009). 18663742 10.1002/ajhb.20805

[b40] von Cramon-Taubadel N. Congruence of individual cranial bone morphology and neutral molecular affinity patterns in modern humans. Am. J. Phys. Anthropol60. 156, 205–215 (2009). 10.1002/ajpa.21041 19418568

[b41] Konigsberg L. W. & Ousley S. D. Multivariate quantitative genetics of anthropometric traits from the Boas data. Hum. Biol. 67, 481–498 (2009). 7541775

[b42] Mosimann J. E. Size allometry: size and shape variables with characterizations of the lognormal and generalized gamma distributions. JASA 65, 930–945 (1970).

[b43] Jungers W. L., Falsetti A. B. & Wall C. E. Shape, relative size, and size adjustments in morphometrics. Yearb. Phys. Anthropol. 38, 137–161 (1995).

[b44] Relethford J. H. & Blangero J. Detection of differential gene flow from patterns of quantitative variation. Hum. Biol. 62, 5–25 (1990). 2323770

[b45] Relethford J. H., Crawford M. H. & Blangero J. Genetic drift and gene flow in post-famine Ireland. Human Biology 69, 443–465 (1997). 9198306

[b46] Wissler C. *The Relation of Man to Nature in Aboriginal North America*. D. Appleton (1926).

[b47] Wissler C. Distribution of moccasin decorations among the Plains tribes. Anthropol. Pap. Am. Mus. Nat. Hist. 29, 5–23 (1927).

[b48] Spier L. The Sun Dance of the Plains Indians: its development and diffusion. Anthropol. Pap. Am. Mus. Nat. Hist. 16, 453–527 (1921).

[b49] Morrow M. Indian Rawhide: An American Folk Art. University of Oklahoma Press (1975).

[b50] Hammer Ø., Harper D. & Ryan P. D. PAST: Paleontological statistics software package for education and data analysis. Palaeontologia Electronica 4, 1–9 (2001).

[b51] Jordan P. & Shennan S. Cultural transmission, language, and basketry traditions amongst the California Indians. J. Anthropol. Archaeol. 22, 42–74 (2003).

[b52] Shennan S. Quantifying Archaeology. Edinburgh University Press (1997).

[b53] Ersts P. J. *Geographic Distance Matrix Generator*. American Museum of Natural History. http://biodiversityinformatics.amnh.org/open_source/gdmg (2016) (Date of access: 15/01/2016).

[b54] Campbell L. American Indian Languages. Oxford: Oxford University Press (1997).

[b55] Hammarström H., Forkel R., Haspelmath M. & Nordhoff S. *Glottolog* 2.3. Max Planck Institute for Evolutionary Anthropology. http://glottolog.org (2014) (Date of access: 15/01/2016).

[b56] Hollow R. C. & Parks D. R. In Anthropology on the Great Plains eds Wood W. R., Liberty M. 68–97 University of Nebraska Press (1980).

[b57] Welsch R. L. Terrell J. & Nadolski J. A. Language and culture on the north coast of New Guinea. Am. Anthropol. 94, 568‒600 (1992).

[b58] Mantel N. A. The detection of disease clustering and a generalized regression approach. Cancer Res. 27, 209–220 (1967). 6018555

[b59] Smouse P. E. & Long J. C. Matrix correlation analysis in anthropology and genetics. Yearb. Phys. Anthropol. 35, 187–213 (1992).

[b60] Rosenberg M. S. & Anderson C. D. PASSaGE: Pattern analysis, spatial statistics and geographic exegesis. Methods in Ecology and Evolution 2, 229–232 (2011).

