# Supplementary material for: Transmission of biology and culture among post-contact Native Americans on the western Great Plains
Source: Sci Rep. 2016 Aug 12;6:25695. doi: 10.1038/srep25695 (PMC4981875; doi:10.1038/srep25695)
Supplement: Supplementary Information [file srep25695-s1.pdf]

Supplementary information for:

Transmission of biology and culture among post-contact Native Americans on the western Great Plains

Stephen J. Lycett[1] and Noreen von Cramon-Taubadel[1]

[1]Department of Anthropology, University at Buffalo, SUNY, 380 MFAC-Ellicott Complex, Amherst, NY 14261, USA

Supplementary Information

Supplementary Table 1: Among-group biometric distance (D-squared) matrix

	Arapaho	Assiniboine	Blackfoot	Cheyenne	Comanche	Crow	Teton	Kiowa	Sarcee
Arapaho	0	0.184888	0.076229	0.069956	0.366093	0.214941	0.087551	0.080705	0.112109
Assiniboine	0.184888	0	0.084419	0.140389	0.414382	0.159373	0.065254	0.201643	0.061857
Blackfoot	0.076229	0.084419	0	0.00694	0.252597	0.120859	0.018263	0.017522	0.01975
Cheyenne	0.069956	0.140389	0.00694	0	0.157372	0.062039	0.010709	0.003564	0.108578
Comanche	0.366093	0.414382	0.252597	0.157372	0	0.079671	0.256683	0.15269	0.35413
Crow	0.214941	0.159373	0.120859	0.062039	0.079671	0	0.080947	0.108781	0.211566
Teton	0.087551	0.065254	0.018263	0.010709	0.256683	0.080947	0	0.059987	0.104846
Kiowa	0.080705	0.201643	0.017522	0.003564	0.15269	0.108781	0.059987	0	0.084394
Sarcee	0.112109	0.061857	0.01975	0.108578	0.35413	0.211566	0.104846	0.084394	0

Supplementary Table 2: Among-tribe cultural distance matrix

	Arapaho	Assiniboine	Blackfoot	Cheyenne	Comanche	Crow	Teton	Kiowa	Sarcee
Arapaho	0	0.63249	0.59222	0.37803	0.56164	0.63895	0.57073	0.62533	0.70027
Assiniboine	0.63249	0	0.58723	0.70138	0.5726	0.65327	0.6317	0.78793	0.6062
Blackfoot	0.59222	0.58723	0	0.61985	0.68143	0.67111	0.66326	0.79912	0.49561
Cheyenne	0.37803	0.70138	0.61985	0	0.52132	0.71832	0.59709	0.64049	0.65209
Comanche	0.56164	0.5726	0.68143	0.52132	0	0.66667	0.47868	0.54795	0.58984
Crow	0.63895	0.65327	0.67111	0.71832	0.66667	0	0.6453	0.76872	0.75647
Teton	0.57073	0.6317	0.66326	0.59709	0.47868	0.6453	0	0.7446	0.71764
Kiowa	0.62533	0.78793	0.79912	0.64049	0.54795	0.76872	0.7446	0	0.75647
Sarcee	0.70027	0.6062	0.49561	0.65209	0.58984	0.75647	0.71764	0.75647	0

Supplementary Table 3: Geographic coordinates for the nine tribes

	LATITUDE	LONGITUDE
Arapaho	39.9	-103.1
Assiniboine	48.99	-103.67
Blackfoot	51.09	-113.77
Cheyenne	43.05	-106.25
Comanche	32.39	-101.25
Crow	46.67	-110.65
T. Dakota	44	-101.5
Kiowa	36.94	-99.03
Sarcee	53.45	-115.22

Supplementary Table 4: Linguistic distance matrix

	Arapaho	Assiniboine	Blackfoot	Cheyenne	Comanche	Crow	Teton	Kiowa	Sarcee
Arapaho	0	5	50	50	5	5	5	5	5
Assiniboine	5	0	5	5	5	50	80	5	5
Blackfoot	50	5	0	50	5	5	5	5	5
Cheyenne	50	5	50	0	5	5	5	5	5
Comanche	5	5	5	5	0	5	5	5	5
Crow	5	50	5	5	5	0	50	5	5
Teton	5	80	5	5	5	50	0	5	5
Kiowa	5	5	5	5	5	5	5	0	5
Sarcee	5	5	5	5	5	5	5	5	0

Supplementary Table 5: Matrix describing intertribal political relationships

	Arapaho	Assiniboine	Blackfoot	Cheyenne	Comanche	Crow	Teton	Kiowa	Sarcee
Arapaho	10	0	0	10	10	-10	10	10	0
Assiniboine	0	10	-10	-10	0	10	-10	0	-10
Blackfoot	0	-10	10	0	0	-10	-10	0	10
Cheyenne	10	0	0	10	10	-10	10	10	0
Comanche	10	0	0	10	10	0	0	10	0
Crow	-10	10	-10	-10	0	10	-10	0	-10
Teton	10	-10	-10	10	0	-10	10	0	-10
Kiowa	10	0	0	10	10	0	0	10	0
Sarcee	0	-10	10	0	0	-10	-10	0	10

-10 = Known hostility; 0 = Little direct/infrequent interaction; 10 = Amicable/known alliance
